# Supplementary material for: Ability of the Right Ventricle to Serve as a Systemic Ventricle in Response to the Volume Overload at the Neonatal Stage
Source: Biology (Basel). 2022 Dec 15;11(12):1831. doi: 10.3390/biology11121831 (PMC9775952; doi:10.3390/biology11121831)
Supplement: Supplementary file 1 [file biology-11-01831-s001.zip › Table S1 Primers.pdf]

Supplemental Table S1 Primer information

Gene		Sequence (5'->3')
MiR-194	Forward	GTCATCACGTGTAACAGCAACT
	Reverse	AGGCCATCAAAAGTAACAGCA
MiR-192	Forward	GTCAAGATGGAGTGCACAGGG
	Reverse	GCATTGAGGCGAACATACCT
MiR-215	Forward	GTGTACAGGACAATGACCTATGATT
	Reverse	ATTGGCCTACAGAATGACAGAC
MiR-221	Forward	GGGCATGAACCTGGCATACA
	Reverse	TAGCCTGAAACCCAGCAGAC
MiR-222	Forward	GTGCCCTCAGTGGCTCAGTA
	Reverse	GATGCCATCAGAGACCCAGTAG
MiR-21	Forward	CCACCTTGTCGGGTAGCTTA
	Reverse	ACCAAAATGTCAGACAGCCCA
Thra	Forward	GGTCACCAGATGGAAAGCGAA
	Reverse	CCTTGTCCCCACACACGAC
Insr	Forward	ATGGGCTTCGGGAGAGGAT
	Reverse	CTTCGGGTCTGGTCTTGAACA
