# Supplementary material for: Ability of the Right Ventricle to Serve as a Systemic Ventricle in Response to the Volume Overload at the Neonatal Stage
Source: Biology (Basel). 2022 Dec 15;11(12):1831. doi: 10.3390/biology11121831 (PMC9775952; doi:10.3390/biology11121831)
Supplement: Supplementary file 1 [file biology-11-01831-s001.zip › Table S2 Reagents.pdf]

Supplemental Table S1 Reagents and antibodies

Name	Company	Catalog No.
Triton X-100	Sigma-Aldrich	T9284
Hematoxylin and eosin Kit	Beyotime biotech	C0105M
DNase	Worthington, Lakewood, NJ, USA	9003-98-9
Type II collagenase	Worthington	9001-12-1
Dispase	Gibco	17105041
RNase	Worthington, Lakewood, NJ, USA	9001-99-4
paraformaldehyde(PFA)	Sigma-Aldrich	158127
4',6-diamidino-2-phenylindole(DAPI)	ThermoFisher Scientific	D3571
Anti-ACSL1 antibody	Abcam	ab177958
PureLink RNA Micro Scale Kit	Life Technologies, Carlsbad, California, USA	12183016
PrimeScriptTM reagent kit	Takara Bio, Kusatsu, Japan	RR037A
SYBR Green Power Premix Kits	Applied Biosystems, Foster City, California	4368577
NEB Next® UltraTM RNA Library Prep Kit	NEB, USA	E7760
Oil Red Stain Kit	abcam	ab150678
Anti-Thyroid Hormone Receptor alpha	abcam	ab53729
Anti-Insulin Receptor beta	abcam	ab278100
TruSeq PE Cluster Kit	Illumina	v3-cBot-HS
