# Supplementary figures and images for: Ability of the Right Ventricle to Serve as a Systemic Ventricle in Response to the Volume Overload at the Neonatal Stage
Source: Biology (Basel). 2022 Dec 15;11(12):1831. doi: 10.3390/biology11121831 (PMC9775952; doi:10.3390/biology11121831)

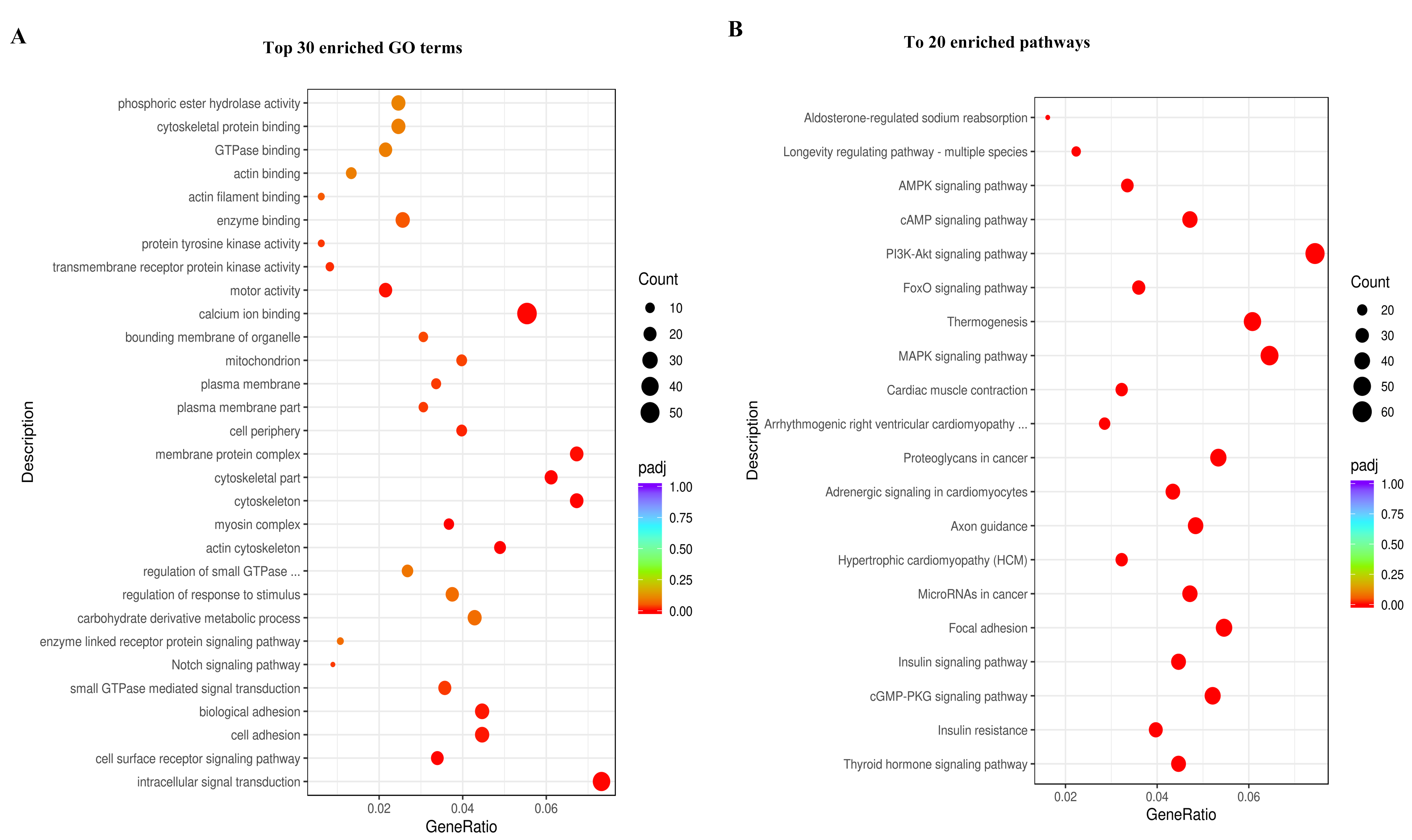

Supplement: Supplementary file 1 [file biology-11-01831-s001.zip › Supplemental Fig. S1-20220926.tif]

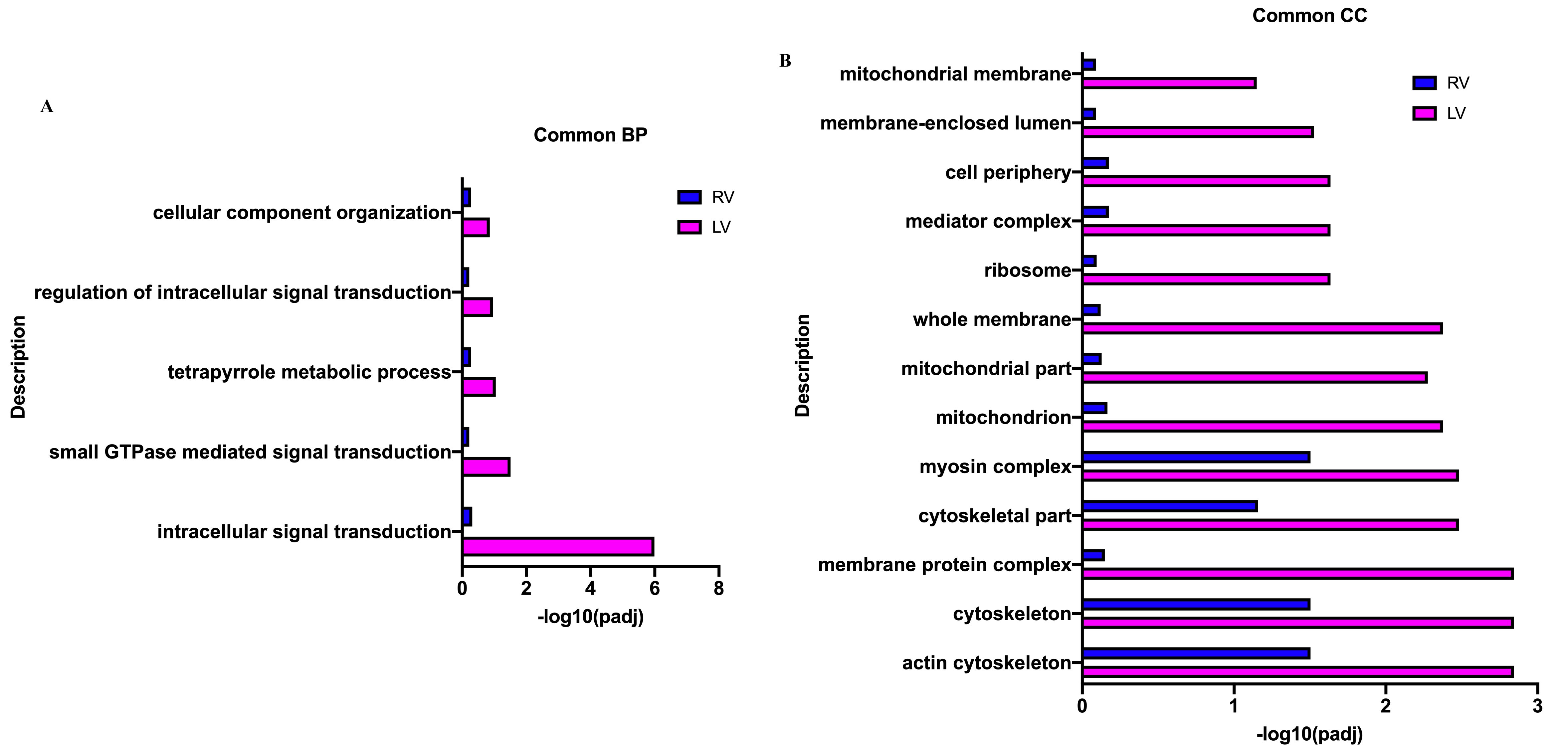

Supplement: Supplementary file 1 [file biology-11-01831-s001.zip › Supplemental Fig. S2-20220926.tif]

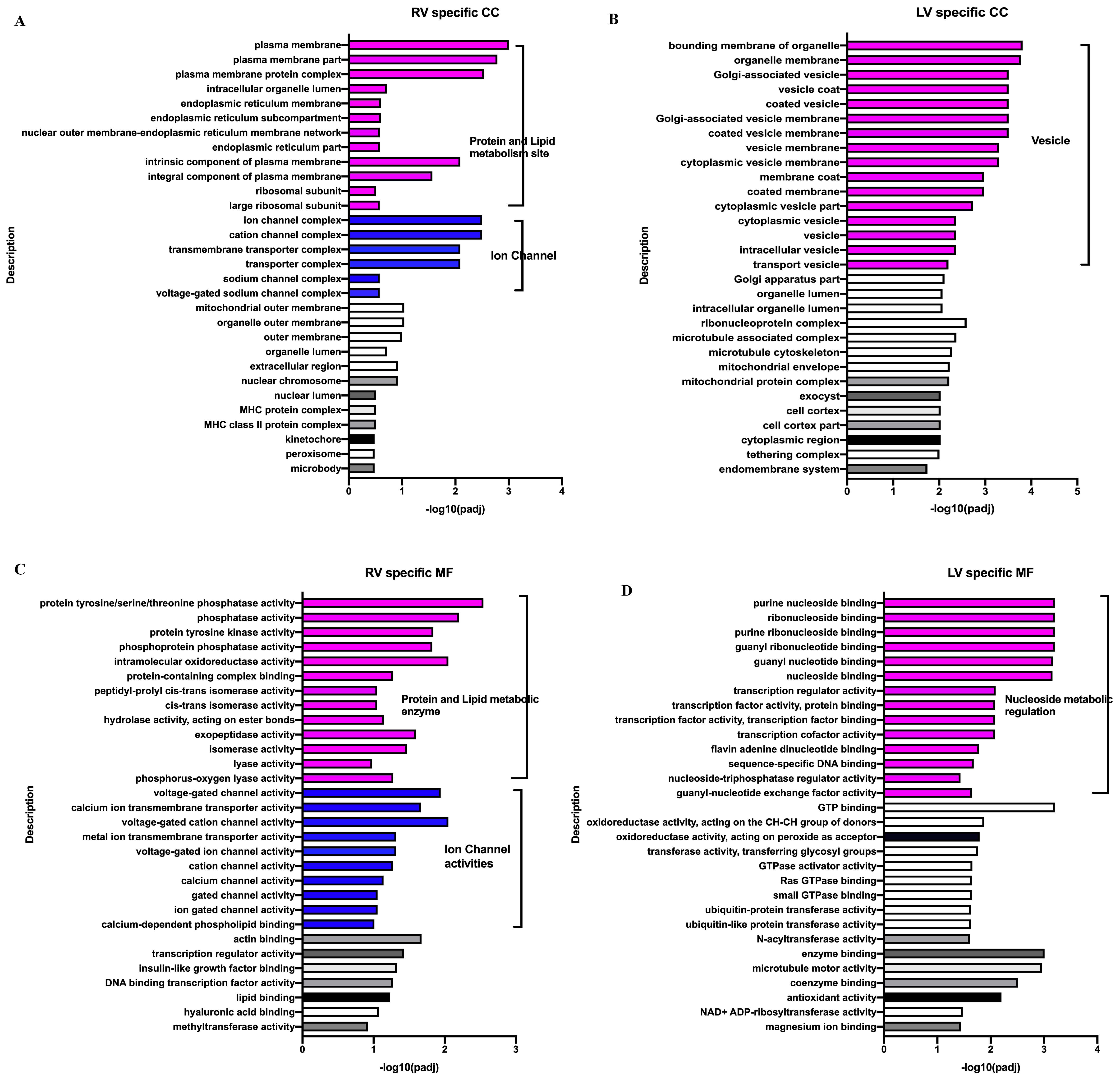

Supplement: Supplementary file 1 [file biology-11-01831-s001.zip › Supplemental Fig. S3-20220926.tif]

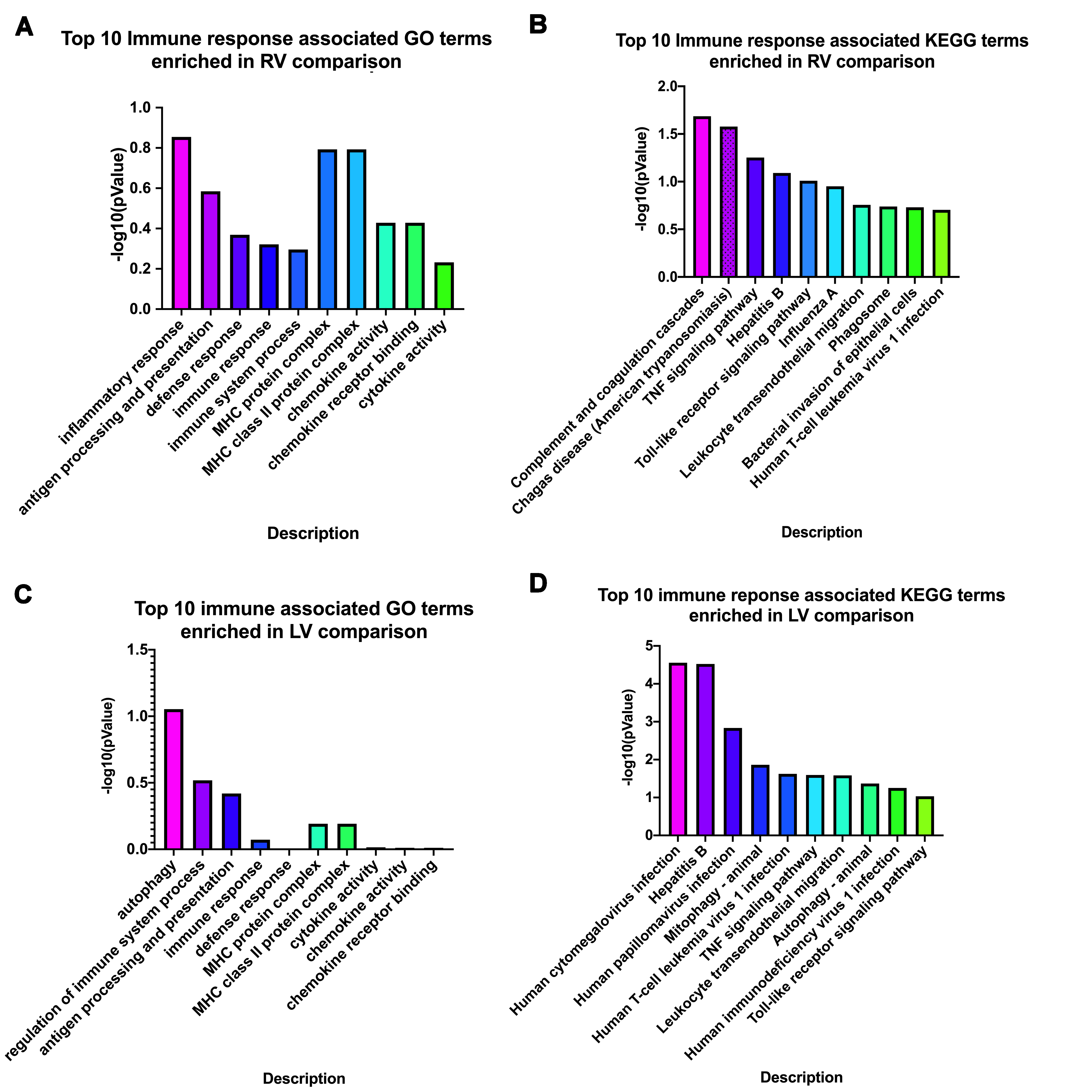

Supplement: Supplementary file 1 [file biology-11-01831-s001.zip › Supplemental Fig.S4-20220810.tif]

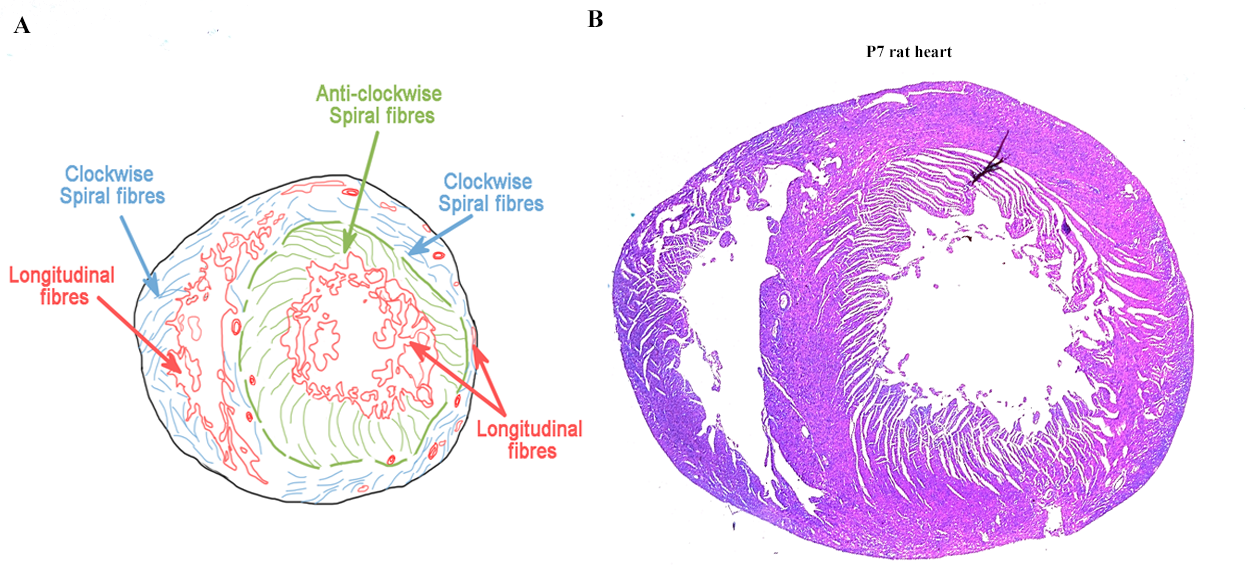

Supplement: Supplementary file 1 [file biology-11-01831-s001.zip › Supplemental Fig.S5-20220926.tif]
